# Specialised ribosomes as versatile regulators of gene expression

**DOI:** 10.1080/15476286.2022.2135299

**Published:** 2022-10-18

**Authors:** Minju Joo, Ji-Hyun Yeom, Younkyung Choi, Hyeon Jun, Wooseok Song, Hyun-Lee Kim, Kangseok Lee, Eunkyoung Shin

**Affiliations:** Department of Life Science, Chung-Ang University, Seoul, Republic of Korea

**Keywords:** Specialised ribosome, ribosome heterogeneity, divergent rRNA, ribosomal protein, post-translational modification, orthogonal ribosome

## Abstract

The ribosome has long been thought to be a homogeneous cellular machine that constitutively and globally synthesises proteins from mRNA. However, recent studies have revealed that ribosomes are highly heterogeneous, dynamic macromolecular complexes with specialised roles in translational regulation in many organisms across the kingdoms. In this review, we summarise the current understanding of ribosome heterogeneity and the specialised functions of heterogeneous ribosomes. We also discuss specialised translation systems that utilise orthogonal ribosomes.

## Introduction

**Figure 1.**
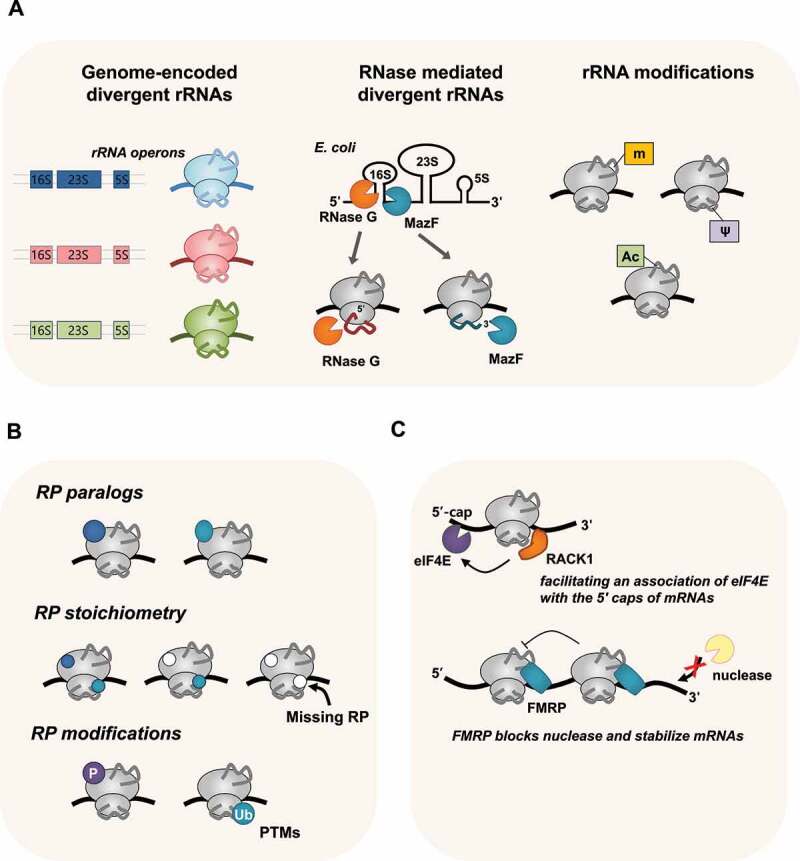
Ribosome heterogeneity in nature. (A) Heterogeneity derived from genome or ribonuclease-mediated divergent rRNA sequences and its modifications can result in different types of ribosomes. (B) Specialised ribosomes also can originate from variations in RPs, including RP paralogs, RP stoichiometry, and PTM of RPs. (C) Unique interactions with ribosome-associated factors, such as RACK1 or FMRP, generate specialised ribosomes in nature.

Ribosomes translate genetic information from mRNAs to proteins. They are fundamental multi-subunit macromolecules comprising ribosomal RNA (rRNA) and ribosomal proteins (RPs). Since the discovery of ribosomes in the mid-1950s, researchers have attempted to elucidate the structure and function of this large ribonucleoprotein complex. Ribosomes consist of two asymmetric subunits, a small subunit (SSU) and a large subunit (LSU); however, the internal composition of these subunits and their macromolecular size vary among organisms. For example, in the yeast *Saccharomyces cerevisiae*, ribosomes are composed of an SSU (40S) with 33 RPs and 18S rRNA and an LSU (60S) with 46 RPs and 3 rRNAs (25S, 5.8S, and 5S rRNA) that together form an 80S ribosome [1–6]. In *Escherichia coli*, 70S ribosomes consist of two subunits, which are designated as the 30S subunit (SSU), comprising 16S rRNA and 21 RPs, and the 50S subunit (LSU), comprising 23S rRNA, 5S rRNA, and 33 RPs [5,7–11]. Both rRNAs and RPs undergo significant post-transcriptional and post-translational modifications, respectively. Despite the sheer complexity of the ribosome, which contains several rRNAs and tens of RPs with a wide array of modifications, it has mostly been considered a homogenous, fixed macromolecular machine with little regulatory capacity [12]. Furthermore, all ribosomes were thought to be constructed according to the same specifications, with no tailored regulation. However, the ribosome compositions in certain organisms are now known to be heterogeneous (for recent reviews, see [13,14]. Several studies provide direct evidence of the heterogeneity of specialised ribosomes (Fig. 1). For example, RNA modifications were revealed to be substoichiometric [15], and differential RP synthesis and composition have been identified [16,17]. Thus, a large body of evidence indicates that ribosomes are much more heterogeneous than conventionally thought. This heterogeneity of ribosomes has inspired researchers to investigate the existence of subpopulations of ribosomes containing altered composition and modification of RPs and/or rRNAs that can conduct specialised functions in mRNA translation. The evidence for functional and physiological roles of heterogeneous ribosomes has been recently emerging although the evidence for heterogeneity is in most cases much stronger [18]. In this regard, ribosomes with a specialised function (‘specialised ribosomes’) were defined to have ‘variations in ribosome composition that influence its activity, thereby changing the output of translation’ [18]. Whether ribosome heterogeneity leads to specialised biological function is still a subject of intense debate in the field [19]. In this paper, we review the literature on the heterogeneity and functional roles of specialised ribosomes and specialised translation systems that utilise orthogonal ribosomes.

### Early speculations on specialised ribosomes

Since the discovery of electron microscopy in 1955, ribosome composition has been thought to vary based on the observation of differences in the size and shape of ribosomes [20,21]. Consistent with this speculation, Dr Francis Crick proposed the ‘one gene-one ribosome-one protein hypothesis’, which postulated that each ribosome contains genetic information (i.e. mRNA) required to encode a single protein [22]. In 1961, this hypothesis was discredited based on observations that showed no generation of new ribosomes when RNA from the bacteriophage genome was synthesised in *E. coli* after infection [12].

In the next two decades, researchers generally assumed that all ribosomes are the same in the cell; thus, the ribosomes were assumed to be passive translation machines. However, the concept of specialised ribosomes became prominent again in the 1980s, because studies indicated the differential expression of individual ribosome components in multiple, diverse invertebrate model organisms. For example, the malaria-causing parasite *Plasmodium berghei* was shown to differentially express two divergent rRNA operons (*rrn*) over different phases of its life cycle [23,24]. In addition, multiple paralogs of RPs are expressed in different regions of *Arabidopsis thaliana* [25,26]. In 2002, the ‘Ribosome Filter Hypothesis’ was suggested [27]. This model suggests that ribosomal composition is a filter that mediates interactions between specific mRNAs and components of the translational machinery [27]. Komili *et al*. also proposed the concept of the ‘ribosome code’ for functionally distinct forms of ribosomes in yeast [28]. Based on these findings, the concept of specialised ribosomes has been suggested as subpopulations of highly heterogeneous ribosomes with the capacity for preferential translation of specific mRNA species, indicating an active part in translational regulation (for recent reviews, see [13,14,29–32]).

### Specialised ribosomes in nature

#### Genome-encoded divergent rRNAs-mediated specialised ribosome

The rRNA operon is generally found in multiple copies, ranging from 1 to 15 copies in bacteria, 1–4 copies in archaea, and thousands in eukarya [33–35]. Phylogenetic analyses of rRNA sequences are based on the assumption that organisms have evolved to possess rRNA genes that express identical and unique rRNA molecules via homogenisation of rRNA genes through gene conversion [36–38]. However, rRNA heterogeneity has been observed in individual organisms in all three domains of life since the 1980s [39–42]. This variability in the rRNA sequences in individual organisms not only poses a problem in the taxonomic assignments of these organisms but also suggests the specialised activity of ribosomes [43].

The first case of rRNA heterogeneity was reported in the malaria parasite *P. berghei* [23,24] (Fig. 1A). The sporozoite form of rRNAs is predominantly found in mosquitoes, whereas the gametocyte form is mainly expressed in the red blood cells of the host after infection. Functional differences between the two types of rRNAs have not been identified; thus, they are suggested to be functionally equivalent [44]. In addition, the context-dependent expression of specific rRNA operons has been documented in bacteria [45], zebrafish [46], and archaea [47]. For instance, *Streptomyces coelicolor*, a model actinomycetes strain, differentially expresses heterogeneous rRNAs during various stages of morphological development [48,49]. The genome of the halophilic archaebacterium *Haloarcula marismortui* contains three 16S rRNA operons: *rrnA, rrnB*, and *rrnC*. The sequence of *rrnC* is virtually identical to that of *rrnA*, whereas *rrnB* shows a high divergence in nucleotide sequences [50,51]. Subsequent studies suggested that divergent copies of the *H. marismortui* rRNA operon can contribute rapid growth over a wide range of temperatures, because of their optimal functional structures at various temperatures [47].

In *E. coli*, there are seven rRNA operons (*rrnA-E, G*, and *H*) with similar but not identical sequences [52,53]. They are located non-contiguously within the genome and are differentially expressed, suggesting that they provide a selective advantage in rapidly adapting to environmental changes via an unknown mechanism [45,54]. Specifically, among these *E. coli* rRNA operons, *rrnH* is highly expressed in response to nutrient limitation-induced stress [54]. The expression levels of ribosomes bearing 16S rRNA from the *rrnH* operon affect the expression of functionally coherent gene sets, including the master regulators of the general stress response, RpoS, and stress-related protein factors, RelA and RelE [54]. Endogenously encoded rRNA sequence variations have also been documented in *Vibrio vulnificus* CMCP6 [55]. Among the divergent rRNAs of *V. vulnificus* CMCP6, ribosomes bearing rRNAs encoded by the *rrnI* operon (I-ribosomes) are responsible for preferential translation of specific mRNAs [56]. This preferential translation by I-ribosomes appears to help *V. vulnificus* cells adapt to environmental changes, such as temperature shifts and variations in nutrient availability. In this case, I-ribosome-mediated preferential mRNA selection appears to use a non-canonical mRNA selection mechanism, not Shine–Dalgarno (SD)-anti-SD (ASD) interactions [56].

The widespread, persistent existence of divergent rRNA genes throughout evolution suggests an adaptive advantage. Further studies are required to dissect the cellular functions of ribosomes containing species-specific genome-encoded divergent rRNAs in other organisms and their evolutionary relationships.

#### Ribonucleases-generated divergent rRNAs-mediated specialised ribosome

Specialised ribosomes bearing heterogeneous rRNA generated post-transcriptionally by ribonucleases have been reported in *E. coli*. The stress-induced free toxin MazF, a sequence-specific endoribonuclease, cleaves target mRNAs specifically at certain single-stranded ACA-sites, leading to the rapid arrest of cell growth [57] (Fig. 1). MazF also cleaves the 3′-end of 16S rRNAs, removing 43 nucleotides from the 3’-end containing the anti-SD sequence, generating 70S^Δ43^ ribosomes [58]. These modified ribosomes were proposed to have the ability to selectively translate leaderless mRNAs generated by MazF. However, the existence and functional role of 70S^Δ43^ ribosomes remain controversial [59,60]. Unlike *E. coli* MazF, a *Mycobacterium tuberculosis* orthologue of MazF (MazF-mt6) can cleave 23S rRNA in the evolutionarily conserved helix/loop 70, resulting in production of modified 50S subunit that is defective in ribosomal subunit association [61]. These findings imply that MazF-mediated heterogenous ribosomes exist although their specialized function in physiological processes need to be further studied.

Another *E. coli* endoribonuclease, RNase G, also participates in the generation of heterogeneous 16S rRNAs in *E. coli* cells when exposed to aminoglycoside antibiotics, such as kanamycin and neomycin [62,63]. Analyses of 16S rRNA from aminoglycoside-resistant *E. coli* cells showed the accumulation of 16S rRNA precursors containing 3–8 extra nucleotides at the 5’ terminus due to incomplete processing by RNase G. A subpopulation of heterogeneous ribosomes bearing incompletely processed 16S rRNA has low affinity for aminoglycoside antibiotics and, consequently, renders *E. coli* cells resistant to these antibiotics [62].

Notably, *rrn* operon disruption (referred to as ‘unlinked *rrn* operons’) is widespread in prokaryotes [64]. In *Helicobacter pylori*, independent maturation of the 16S and 23S-5S precursors that were separately transcribed from the unlinked *rrn* operon was observed [65]; which probably underwent different processing pathways, resulting in the generation of divergent rRNA species. Most bacteria with unlinked-*rrn* operons are symbiotic bacteria, indicating specific roles of rRNA species transcribed from them in symbiosis [66].

These studies suggest that subpopulations of ribosomes bearing heterogeneous rRNAs generated by differential processing have important biological roles in response to various stress conditions.

#### Chemically modified rRNAs-mediated specialised ribosome

In all organisms, rRNAs undergo extensive post-transcriptional modifications that facilitate the diversity of their composition and ribosome activity (Fig. 1A). To date, in human 80S ribosomes, rRNA contains 228 sites with 14 distinct types of post-transcriptional modifications [67], whereas there are 36 modified nucleotides in *E. coli* rRNAs [68]. Among the types of rRNA modifications, methylation on the 2-hydroxyl group of ribose (2ʹOme) and the conversion of uridine to pseudouridine (Ψ) are the most abundant modifications in eukaryotes [69]. These modifications can contribute to ribosome heterogeneity because a subset of positions is fractionally modified, and rRNA modifications are identified at substoichiometric amounts [70,71].

In many cases, the precise roles of rRNA modifications are largely unknown. However, many of these modifications are conserved and clustered in functionally important regions of rRNAs, such as the peptidyl transferase centre (PTC) or ribosomal subunit interface [72,73]. Thus, their potential functions in catalytic activity and ribosomal structure have been proposed [72,74–76]. In this regard, a study showed that the hydroxylation of I5 at position 2501 of 23S rRNA is in close proximity to the PTC fine-tunes translation and provides a growth benefit to *E. coli* under oxidative stress [77]. In yeast, ribosomes have five modifications. The loss of three to five modifications in the inter-subunit bridge (helix 69) substantially impairs cell growth due to altered rRNA structure, suggesting that a subset of rRNA modifications can influence ribosome assembly and function synergistically [78]. Additionally, some rRNA modifications affect the susceptibility of ribosomes to antibiotics, leading to antibiotic resistance [79–82].

Uneven rRNA modifications, such as partial and altered modification patterns, further imply the existence of additional roles under certain physiological conditions [70,83–85]. Although it remains unclear how rRNA modifications are dynamically regulated under different physiological conditions, these chemical modifications exert a cumulative effect on ribosome activity and, in turn, confer advantages in response to environmental changes.

#### RP paralog-mediated specialised ribosome

Ribosome heterogeneity may also originate from the incorporation of different core RP paralogs (Fig. 1B). RPs are highly conserved across all forms of life (for a relevant review, see [86]); at least 53 and 80 RPs have been detected in *E. coli* and eukaryotic ribosomes, respectively [4,87,88]. RPs have been shown to impart selectivity to translating ribosomes, implicating them in gene expression control. In the Amoeba *Dictyostelium discoideum*, significant changes in RP composition were observed at various stages of development. Twelve distinct RPs appear at specific stages of cell differentiation (between vegetative amoebae and spores). Among them, two RPs were specific to ribosomes of vegetative amoebae, and three were specific to spores, suggesting that RP paralogs may contribute to the different regulation of protein synthesis during growth and development [89]. In *S. cerevisiae*, 59 of the 78 RPs retain two genomic copies [90]. Notably, these duplicated RP paralogs have nearly identical amino acid sequences and similar patterns of transcriptional regulation, but the knockout of individual RP paralogs results in different phenotypes [28]. In a study on well-characterised *ASH1* mRNA in yeast, Komili *et al*. showed that duplicated RPs have distinct functional roles in translational regulation, including ribosomal assembly and paralog-specific aberrant localisation [28]. In addition, the RP paralogs eL8A and eL8B change their relative stoichiometry in the population of ribosomes when yeast cells are grown in different carbon sources, suggesting that ribosomes can alter their composition and functional activity in response to changes in growth or environmental conditions [17].

Approximately 46.5% of bacterial genomes have paralogous genes for one to three RPs [91]. Some RPs, such as bL31, bL33, bL36, and uS14, are duplicated in more than 100 bacterial genomes. Comparative bacterial genomic data indicate that several RPs are encoded by multiple paralogous genes, suggesting the structural heterogeneity of ribosomes. In *E. coli*, two ribosomal core proteins, bL31 and bL36, have two paralogs: bL31A and bL31B, and bL36A and bL36B, respectively [92]. A study showed the composition of these two RPs (bL31 and bL36) during different bacterial growth phases in *E. coli* [93]. In the exponential phase, most ribosomes contain bL31A and bL36A paralogs, whereas in the stationary phase, bL31B and bL36B are prevalent paralogs. In addition, ribosomes with bL31A conferred higher compatibility with *E. coli* at lower temperatures and maintained the translation reading frame more efficiently than ribosomes with bL31B *in vivo* [94]. In addition, ribosomes containing uL1 facilitate the efficient translation of respiration-related proteins in yeast, suggesting that the heterogeneity of RP paralogs defines a novel means of translational control [32].

Tissue-specific expression of paralogs of core RP has also been observed at the transcript and protein levels [95]. For example, in *Drosophila melanogaster*, some RP paralogs show differential expression in the adult male germline [96]. eL22 is transcribed ubiquitously, whereas eL22‑like (dL22L) is predominantly expressed in the testes. Paralogs of uL16, eL22, and eL39 are also differentially expressed in mice [97]. uL16L and eL39L are only found in ribosomes from the testis but not in the liver or mammary glands [97]. Similar observations have been reported for the tissue-specific expression of RP paralogs in humans. eL39L is highly expressed in cancer cell lines, and its expression is strongly correlated with vascular invasion in hepatocellular carcinoma tumour samples [95].

Further studies are required to identify the functional redundancy or specificity of core RP paralogs and how they act jointly to create a specialised ribosome.

#### RP stoichiometry-mediated specialised ribosome

Another possibility for creating heterogeneous ribosomes is to alter the relative abundance of core RPs (Fig. 1B). Ribosomes were assumed to have a fixed stoichiometry among their core RPs for proper protein synthesis function [98–100]. However, this notion has been challenged by multiple observations that under distinct conditions, as well as in diverse cell types, some RPs are present in substoichiometric amounts on ribosomes [89,101], indicating that a heterogeneous RP complement can alter translational activity. Recent data provided direct evidence for alterations in stoichiometry in isolated ribosomes with high specificity and accuracy throughput (for a recent review, see [101]). Differential stoichiometry was found in the core RPs in budding yeast *S. cerevisiae* and mouse embryonic stem cells (mESCs), suggesting that stoichiometry among core RPs depends on both growth conditions and the number of ribosomes bound per mRNA [102]. These findings provide further evidence of specialised ribosomes with distinct RP compositions and related physiological functions.

Furthermore, ribosomes lacking specific RPs are present in cells. For instance, using the selected-reaction monitoring (SRM)-based MS method, the absolute abundance of RPs in translating ribosomes and profiled transcripts enriched or depleted from subsets of ribosomes were measured in mouse ESCs [103]. In this study, four RPs (uL1, eL38, eS7, and eS25) were at significantly substoichiometric levels in mouse ESCs, revealing that there are actively translating ribosomes lacking at least one core RP.

These findings indicate that alterations in the RP composition can result in ribosome heterogeneity, contributing to translational control.

#### Post-translational modification (PTM) of RP-mediated specialised ribosomes

Heterogeneity in ribosomes due to the PTM of RPs, including phosphorylation, ubiquitination, methylation, acetylation, and SUMOylation, has been postulated to generate ‘specialised ribosomes’. In many organisms, most RPs are post-translationally modified. In *E. coli*, comparative proteomic analysis has revealed that six RPs were methylated (uS11, uL3, uL11, bL12, uL16, and bL33), three proteins were acetylated (uS5, bS18, and bL12), and protein uS12 was methylthiolated [104,105]. Several RPs are differentially acetylated or phosphorylated during the exponential or stationary growth phase of *E. coli* [106,107]. Notably, acetylation of bL12 in *E. coli* varies with cell growth and nutrient deprivation, increasing the stability of the ribosomal stalk complex under stress [108]. In yeast, several modifications, such as methylation, acetylation, and hydroxylation of RPs, are highly abundant [109]. For example, uL3, a highly conserved ribosomal protein, is methylated at histidine 243 (H243) by Hpm1 methyltransferase, and this modification plays a key role in translation elongation [110]. In *A. thaliana*, 23 of the 80 cytosolic RP families contain residue-specific covalent modifications that represent potential differential modification sites [111]. The extent of protein phosphorylation in cytosolic ribosomes isolated from the leaves of *A. thaliana* showed an increase in the day/night phosphorylation ratio of RPs, suggesting that differential phosphorylation of RPs may contribute to the modulation of diurnal protein synthesis in plants [112]. Patterns of covalent modifications, including initiator methionine removal, N-terminal acetylation, and phosphorylation, also appear to be highly conserved among plants, animals, and fungi, suggesting that many of these modifications are fundamentally important for ribosomal functions. All these findings support the idea that the PTM of RPs can result in ribosome heterogeneity and, possibly, contribute to the specificity of the translational machinery.

#### Ribosome-associated factors-mediated specialised ribosomes

In addition to the composition of rRNAs and core RPs, the heterogeneity of ribosomes can result from other protein factors associated with the ribosome (Fig. 1C). Proteomic studies with advanced MS-based methods have revealed 77 uncharacterised open reading frames (ORFs) as putative ribosome-interacting factors in *S. cerevisiae* [113]. Cells without these proteins show altered polysome profiles, decreased protein synthesis rates, and translation fidelity, implying that many additional ribosome-associated factors may modulate ribosome activity.

RACK1, the receptor for activated protein C kinase 1, is a highly conserved scaffold protein that can interact with several signalling molecules, either directly or as part of a larger complex (for a review, see [114,115]). Cryo-EM studies have revealed that RACK1 is located on the back of the 40S subunit near the mRNA exit channel and directly contacts rRNA [115,116]. In addition, Asc1, a homologue of RACK1, is necessary for the efficient translation of mRNAs with short ORFs in *S. cerevisiae* [117]. Furthermore, RACK1 and the ubiquitin ligase ZNF598 play pivotal roles in the ribosome-associated quality control pathway by resolving poly(A)-mediated stalled ribosomes and ribosomal ubiquitination in mammalian cells [118,119]. The binding of RACK1 to ribosomes is essential for the translation of capped mRNAs and efficient recruitment of eukaryotic initiation factor 4E (eIF4E) [120]. In addition, depletion of RACK1 or expression of mutant RACK1 defective in ribosome binding appear to alter the ability of the ribosome to translate specific mRNAs [121]. These studies suggest a role for RACK1 as a ribosome-associated factor in transcript-specific translational regulation.

Fragile X mental retardation protein (FMRP) is a well-known ribosome-associated factor. FMRP is a broadly expressed RNA-binding protein that associates with mRNAs and other proteins to form large ribonucleoprotein complexes (mRNPs). These complexes have been proposed to participate in the transport, localisation, and translation of target mRNAs [122]. FMRP inhibits translation by binding directly to the L5 protein on the 80S ribosome and interfering with the binding of translation elongation factors or tRNA to the ribosome [123]. There have been several reports of FMRP-mediated translational regulation [124–127]. FMRP regulates ribosome stalling on specific mRNAs (e.g. SETD2) [126]. In addition, FMRP preferentially binds to mRNAs with optimal codons, suggesting that it stabilises such transcripts through direct interactions via the translational machinery [127].

In *D. melanogaster*, the apoptotic regulator, Reaper, inhibits translation initiation by directly and specifically binding to the 40S ribosomal subunit. Specifically, the direct binding and regulation of ribosome function by Reaper allows the selective translation of mRNAs initiating at alternative start codons or from certain internal ribosome entry site elements [128]. In humans, glycogen synthase 1 (GYS1) is associated with the active elongation of ribosomes. Depletion of intracellular GYS1 results in a loss of polysomes and alters the abundance and translational efficiency of a subset of mRNAs, indicating that GYS1 can control the translation of subsets of mRNAs [129]. These findings indicate how ribosome-associated factors can add another layer of ribosome diversity and specificity.

### Synthetic specialised ribosomes: orthogonal ribosomes

**Figure 2.**
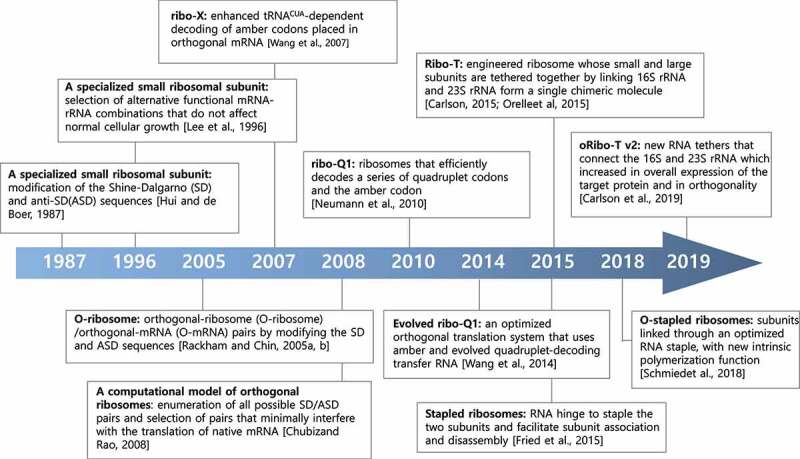
Timeline of key discoveries and developments regarding orthogonal special ribosomes research.

**Figure 3.**
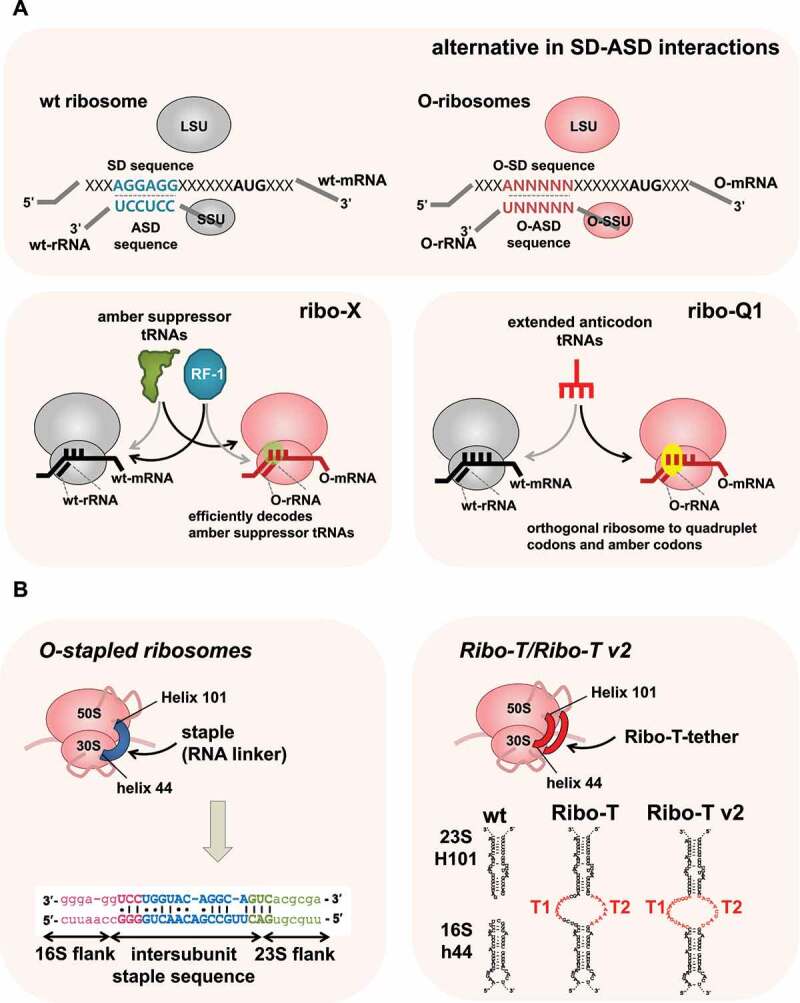
Evolution of orthogonal special ribosomes. (A) Early orthogonal specialised ribosomes were generated by random mutagenesis in the RNA-binding sequences (RBS) on mRNA and its complementary message-binding sequence (MBS) on rRNA in *E. coli* [134]. Orthologue ribosomes have been further evolved to increase the *in vivo* efficiency of unnatural amino acid incorporation (ribo-X [139]) and enable efficient incorporation of amber and quadruplet codons (ribo-Q1 [141,142].(B) The engineering of the fully orthogonal ribosomes became possible with the advent of the ribosome covalently linked through an optimised RNA staple (O-stapled ribosomes [147,148]) or tethered ribosomal subunits with RNA linker (Ribo-T/Ribo-T v2 [146–149];.

In the process of bacterial translation initiation, base pairing between an SD sequence in the 5’ untranslated region of mRNA and a complementary ASD sequence at the 3’ end of 16S rRNA plays a crucial role in mRNA selection by ribosomes [130] (Fig. 2). In 1987, in a pioneering study, Hui and DeBoer created a specialised small ribosomal subunit by modifying the SD sequence of an mRNA and the corresponding pyrimidine-rich ASD sequence in the 16S rRNA [131–133]. They developed a specialised ribosome system by modifying the SD sequence in a cloned copy of the heterologous human growth hormone (hGH) gene from 5’ GGAGG to 5’ CCTCC or 5’ GTGTG, and the ASD sequence of an rRNA operon expressed from the same plasmid to complementary sequences from 5’ CCTCC as ribosomes bearing plasmid-encoded mutant 16S rRNA that can selectively recognise and produce human growth hormone from the hGH mRNA in *E. coli* [131]. Further studies by Lee et al. have shown that modifications of the ASD sequence resulted in cell lysis, probably due to an interruption in the proteome profile caused by enhanced protein synthesis by specialised ribosomes from certain mRNA species that are normally not efficiently recognised by WT ribosomes [134] (Fig. 3A). They generated a random pool of 5,049 combinations of SD-ASD sequences and screened for alternative combinations that did not interfere with normal protein synthesis and produced target proteins with high selective discrimination [134]. These studies show that specific sequence constraints exist for translation in the formation of efficient SD-ASD interactions [134]. An alternative SD-ASD sequence has been used to construct a genetic system to study the functional and structural interrelationships of highly conserved regions of 16S rRNA [135,136]. Furthermore, this alternative SD-ASD sequence has been used to construct specialised ribosome systems, the so called ‘orthogonal ribosome systems’, for following studies by other research groups.

For selectively and preferentially producing recombinant proteins of interest in *E. coli*, highly active and specific orthogonal-ribosome (O-ribosome)/orthogonal-mRNA (O-mRNA) pairs were developed by gene duplication, followed by a new positive and negative selection method [137,138]. This study produced complex synthetic networks to predict interactions between O-ribosomes and O-mRNAs and showed that O-ribosomes exclusively translate orthogonal mRNA not recognised by normal cellular ribosomes [137]. To increase the *in vivo* efficiency of unnatural amino acid incorporation, Wang *et al*. evolved an orthogonal ribosome (ribo-X) that enhanced tRNA_CUA_-dependent decoding of amber codons placed in orthogonal mRNA in *E. coli* [139] (Fig. 3A). This system combines ribo-X with orthogonal mRNAs and orthogonal aminoacyl-tRNA synthetase/tRNA_CUA_ pairs to substantially increase the efficiency of site-specific unnatural amino acid incorporation in *E. coli*. This study enabled the synthesis of proteins that incorporate unnatural amino acids at specific sites *in vivo*. Chubiz and Rao developed a computational model of orthogonal ribosomes by enumerating all possible SD/ASD pairs and then selecting those that minimally interfered with the translation of native mRNA without toxic effects on the cell [140].

Orthologue ribosomes have been further evolved to enable the efficient incorporation of multiple distinct non-canonical amino acids into polypeptides. For example, Neumann et al. synthesised ribo-Q1, which enhanced its efficiency for the translation of quadruplet codons and amber codons, enabling the efficient site-specific incorporation of multiple distinct unnatural amino acids into a single polypeptide [141,142] (Fig. 3A). However, these engineered ribosomes have been limited to the 30S small subunit because 50S large subunits freely exchange between the native and orthogonal 30S small subunits [143].

In overcoming these limitations, engineered ribosomes with tethered subunits, called Ribo-T, have been constructed and subsequently improved by several groups [144–147] (Fig. 3B). Ribo-T is an engineered ribosome whose small and large subunits are tethered by physically linking 16S rRNA and 23S rRNA to form a single chimeric molecule. Chimeric rRNA was created by connecting the 23S rRNA termini within the loop of helix 101 (H101) to the apex loop of 16S rRNA helix 44 (h44) with short RNA linkers [146]. Ribo-T supported the growth and proliferation of *E. coli* cells, even in the absence WT ribosomes, indicating that tethered ribosomes are fully functional.

Other studies have reported similar results with analogously designed ribosomes with conjoined subunits. For example, engineered orthogonal ‘stapled’ (O-stapled) ribosomes, whose subunits are covalently linked through an optimised RNA staple, were discovered to have a new intrinsic polymerisation function [147,148] (Fig. 3B). Carlson et al. developed an improved orthogonal Ribo-T (oRibo-T v2) that functions in parallel with natural ribosomes and mRNAs, increasing the efficiency of orthogonal protein expression [149] (Fig. 3B). Other RNA tethers that connect the 16S and 23S rRNA genes were searched using tether libraries varying in both the length and composition of the tether sequence. These approaches provide a promising strategy to control the association of ribosomal subunits and direct both subunits to an orthogonal message, enabling the evolution of new large-subunit functions that have not been accessed in natural ribosomes. Orthogonal ribosome systems can be used not only to produce recombinant proteins with synthetic components but also for exploring novel and canonical functions of ribosomes *in vivo*.

## Conclusions

Over the past few decades, evidence of gene expression regulation by specialised translation machinery has accumulated. In this review, we focused on heterogeneity in ribosome composition and its functional roles, as well as engineered orthogonal specialised ribosome systems. Although the biological roles of most naturally occurring specialised ribosomes are largely unknown, technological advances in high-throughput analyses and genetic engineering methods combined with bioinformatic analyses would improve the accurate assessment of ribosome heterogeneity and its physiological role. For example, high-resolution native MS analysis provided sufficient resolution to identify differences in the copy number of uL7 and uL12 RPs on intact *E. coli* ribosomes, revealing structural details and providing insights into their composition [150]. Shi et al. employed SRM to measure the absolute abundance of a subset of core RPs and heterogeneous compositions in mESCs [103]. High-throughput MS-based strategies, metabolic labelling approaches such as stable isotope labelling with amino acids in cell culture, and isobaric labelling approaches such as tandem mass tags have been used to quantify the relative abundance of core and ribosome-associated proteins [151]. Single-particle cryo-EM analysis has also been used to quantitatively characterise the significant structural heterogeneity of ribosomes [152].

Although the functional consequences of differences in ribosome composition remain challenging, reports based on these advanced technologies provide compelling evidence in support of the specialised function of ribosomes in various organisms. For example, studies on genome-encoded divergent rRNAs in bacteria have demonstrated that ribosomes bearing these divergent rRNAs modulate the expression of genes that contribute directly to bacterial stress adaptation, highlighting a novel function of specialised ribosomes [54,56]. Further studies will unveil the biological significance of ribosome heterogeneity. For instance, orthogonal ribosome systems can be used to investigate the relationship between ribosome heterogeneity and specialised functions.

Significant progress has been made in the research on engineered specialised ribosomes through functionally orthogonal ribosomes and mRNA *in vivo* to produce recombinant proteins and explore the poorly understood functions of the ribosome. These studies have developed methods for parallel genetic circuits [137] and the incorporation of diverse polypeptides by using non-canonical amino acids [139], expanded genetic codes incorporating amber and quadruplet codons [141,142], and tethered ribosomal subunits with an RNA linker to improve cellular orthogonality [146–149]. Despite the rapid progress in engineered ribosomes, including evolutionary approaches to tethering two subunits of ribosomes, several critical challenges have not been addressed. First, in many cases, they exhibit a diminished rate of protein synthesis or an insufficient growth rate compared with those with WT ribosomes [146]. Second, the unusual structure and transcriptional sequence of rRNA segments in tethered ribosomes cause notable assembly defects [144]. For example, Ribo-T maturation could be additionally affected by an imbalanced production of r-proteins and assembly factors due to the altered functionality of the translation apparatus in Ribo-T cells [144]. Extensive efforts to construct truly orthogonal translation systems are expected to provide a versatile tool for elucidating fundamental questions of ribosomes in nature regarding their origin, evolution, and function, and their efficient production of recombinant proteins.

## Data Availability

Data sharing is not applicable to this article as no new data were created in this study.
